# An Integrative Analysis of Transcriptomics and Proteomics Reveals Novel Insights into the Response in the Midgut of *Spodoptera frugiperda* Larvae to Vip3Aa

**DOI:** 10.3390/toxins14010055

**Published:** 2022-01-13

**Authors:** Minghui Jin, Yinxue Shan, Yan Peng, Ping Wang, Qi Li, Songmiao Yu, Lei Zhang, Yutao Xiao

**Affiliations:** 1 Shenzhen Branch, Guangdong Laboratory of Lingnan Modern Agriculture, Genome Analysis Laboratory of the Ministry of Agriculture and Rural Affairs, Agricultural Genomics Institute at Shenzhen, Chinese Academy of Agricultural Science, Shenzhen 518120, China; jinminghui@caas.cn (M.J.); shanyinxue9@163.com (Y.S.); pengyan0927@gmail.com (Y.P.); wangping2755@163.com (P.W.); liqi9150@163.com (Q.L.); ysm18845726086@163.com (S.Y.); 2 College of Plant Science and Technology, Huazhong Agricultural University, Wuhan 430070, China

**Keywords:** Vip3Aa, *Spodoptera frugiperda*, transcriptomic, proteomic, apoptosis, MAPK

## Abstract

The insecticidal Vip3 proteins, secreted by *Bacillus thuringiensis* (*Bt*) during its vegetative growth phase, are currently used in Bt crops to control insect pests, and are genetically distinct from known insecticidal Cry proteins. Compared with Cry toxins, the mechanisms of Vip3 toxins are still poorly understood. Here, the responses of *Spodoptera frugiperda* larvae after Vip3Aa challenge are characterized. Using an integrative analysis of transcriptomics and proteomics, we found that Vip3Aa has enormous implications for various pathways. The downregulated genes and proteins were mainly enriched in metabolic pathways, including the insect hormone synthesis pathway, whereas the upregulated genes and proteins were mainly involved in the caspase-mediated apoptosis pathway, along with the MAPK signaling and endocytosis pathways. Moreover, we also identified some important candidate genes involved in apoptosis and MAPKs. The present study shows that exposure of *S. frugiperda* larvae to Vip3Aa activates apoptosis pathways, leading to cell death. The results will promote our understanding of the host response process to the Vip3Aa, and help us to better understand the mode of action of Vip3A toxins.

## 1. Introduction

*Bacillus thuringiensis* (*Bt*) can produce insecticidal proteins that are toxic to a number of agricultural pests, and has become the most economically successful entomopathogen to date [1]. Insecticidal crystal (Cry) proteins are produced during its sporulation phase, and have been used to control insect pests through either traditional spraying or transgenic crops [2]. Cry toxins are generally recognized as pore-forming toxins, and the mode of action of these toxins involves solubilization of the protoxin by digestive enzymes, binding to specific receptors located on the midgut BBMV, and inducing pores in the membrane, eventually leading to insect death [3]. However, with the extensive application of Cry toxins, insect resistance cases are also increasing [3].

The vegetative insecticidal proteins (Vips) produced by Bt during its vegetative stages share no sequence homology with known Cry proteins. Vips also display a wide spectrum of insecticidal activity to lepidopteran pests [4,5]. Vip3A and Cry proteins have different insecticidal processes, recognizing different receptors, indicating that Vip3A is likely to complement the toxic action of Cry proteins, reducing the risk of the development of insect resistance in the field. Transgenic corn co-expressing Vip3A and Cry1Ab showed excellent control of target pests, and no cross-resistance between Cry proteins and Vip3A was reported [6,7]. Compared with Cry proteins, much less is known about the mode of action of Vip3A. Previous studies showed that the Vip3A protoxin is activated by proteases in the midgut, yielding an active toxin (~62 kDa) together with the ~20 kDa fragment. The processed active toxin binds to its specific receptors in the midgut and forms pores [8]. Although Vip3A showed similar mode of action to Cry toxins, they do not share membrane-binding sites. Recently, some proteins interacting with Vip3Aa, showing close relation in terms of cell toxicity in *Sf9* cells, were identified, such as S2, scavenger receptor-C, and fibroblast growth factor receptor [9,10,11]. The SRC protein has been reported to be able to mediate Vip3Aa endocytosis, in association with its insecticidal activity [10]. However, in addition to the reported pore-forming model, other mechanisms that induce cell death have also been observed. Jiang et al. [12] found that the Vip3Aa-treated *Sf9* cells had some apoptotic characteristics. Hernandez-Martinez et al. [13] demonstrated that Vip3Aa could induce apoptosis in *Spodoptera exigua* midgut epithelial cells. Apoptosis is indispensable to the homeostasis and development of organisms, and has been described as a mechanism of cellular response after exposure to Bt toxins [14].

*Spodoptera frugiperda*—a noctuid polyphagous lepidopteran pest—causes serious damage to the crops, such as maize, cotton, rice, and vegetables [15]. This pest is native to America, and invaded Africa in 2016 and India in early 2018. Most recently, this pest was detected in Yunnan Province, China, in December 2018 [16]. Currently, the presence of *S. frugiperda* has been confirmed in most Asian countries, as well as in Australia. Bt crops are an important strategy used to control *S. frugiperda*. Transgenic maize events expressing the Vip3Aa insecticidal protein were released for commercial use in Brazil in 2009 [17]. However, with the high adoption rate of Bt maize and the intense crop production system, field-evolved Vip3Aa resistance has occurred rapidly in Brazil and the USA [17,18].

In this study, we combined the transcriptome and proteomic analysis to examine and compare the effects of Vip3Aa treatment in the midgut of *S. frugiperda*. Our goal was to identify the candidate genes involved in the response to Vip3Aa, as well as providing a broad overview of important pathways associated with the mode of action of Vip3Aa.

## 2. Results

### 2.1. Vip3Aa Significantly Inhibited the Growth of S. frugiperda

**Figure 1:**
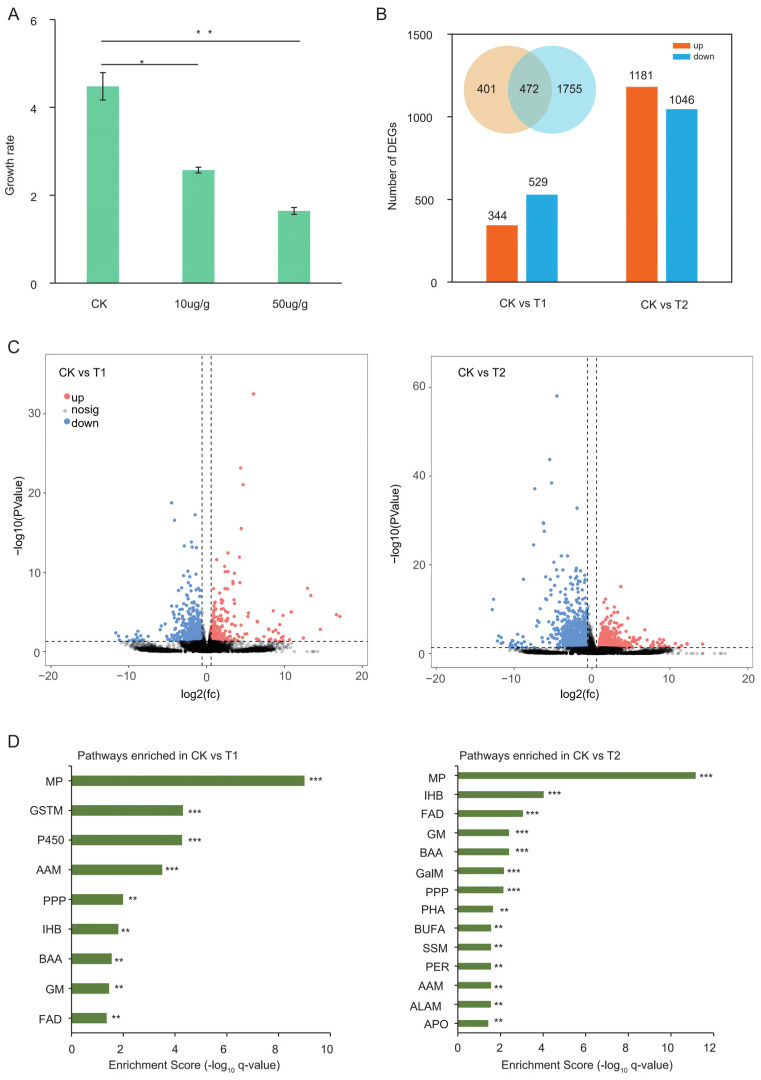
Transcriptomic analysis of Vip3A treatment: (**A**) The relative growth rates of *S. frugiperda* larvae feeding on artificial and Vip3Aa-containing diets; * *p* < 0.05; ** *p* < 0.01. (**B**) The numbers of differentially expressed genes in treatment 1 (10 μg/g, T1) and treatment 2 (50 μg/g, T2); Venn diagram shows the common genes between T1 and T2. (**C**) Volcano plots of CK vs. T1 (**left**) and CK vs. T2 (**right**); *x*-axis represents the ratio of fold changes. (**D**) KEGG pathways enriched in comparison group CK vs. T1 and comparison group CK vs. T2; *x*-axis represents the enrichment score. ** *q* < 0.05; *** *q* < 0.01. Abbreviations—MP: metabolic pathway; GSTM: glycine: serine and threonine metabolism; AAM: ascorbate and aldarate metabolism; PPP: pentose phosphate pathway; IHB: insect hormone biosynthesis; BAA: biosynthesis of amino acids; GM: glutathione metabolism; FAD: fatty acid degradation; GalM: galactose metabolism; PHA: phagosome; BUFA: biosynthesis of unsaturated fatty acids; SSM: starch and sucrose metabolism; PER: peroxisome; ALAM: alpha-linolenic acid metabolism; APO: apoptosis.

*S. frugiperda* larvae (third-instar larvae) were fed on an artificial diet containing two different concentrations of Vip3Aa for three days. The relative growth rates were calculated using larval weight data. Larvae fed on a Vip3Aa diet gained significantly less weight than the larvae fed on a non-toxin-added artificial diet (*p*-value < 0.05) (Figure 1A).

### 2.2. Transcriptomic Analysis of Midguts from Different Treatments

To obtain a comprehensive overview of the mechanism of Vip3Aa and its effect on the growth of *S. frugiperda*, the expression levels of genes from the midguts of controls were compared to those from Vip3Aa treatments. The mapping data analysis revealed that an average of 82.4% clean reads covered the reference genome. A threshold of fold-change ≥ 1.5 and *p*-value < 0.05 was used to judge significant differentially expressed genes (DEGs). The results showed that 344 genes were upregulated and 529 genes were downregulated in treatment 1 (10 μg/g; T1), whereas more differentially up/downregulated genes were identified in treatment 2 (50 μg/g; T2) (Figure 1B,C, Tables S1 and S2). These results indicate that the larvae respond more strongly to the stimulus of higher concentrations of Vip3Aa. A total of 472 genes were commonly regulated in T1 and T2 (Figure 1B). In order to better understand the functional categories, KEGG analysis was performed using DEGs. KEGG results showed that 9 and 14 pathways were enriched in T1 and T2, respectively (Tables S3 and S4). Insect hormone biosynthesis (IHB) was commonly enriched in T1 and T2. However, when the concentration of Vip3A increased, the pathway of apoptosis (APO) was enriched in T2 (*p*-value = 0.0074) (Figure 1D), which is consistent with previous reports that Vip3Aa could exert cytotoxicity by triggering apoptosis, in addition to forming pores [12,14].

### 2.3. Gene Set Enrichment Analysis (GSEA)

**Figure 2:**
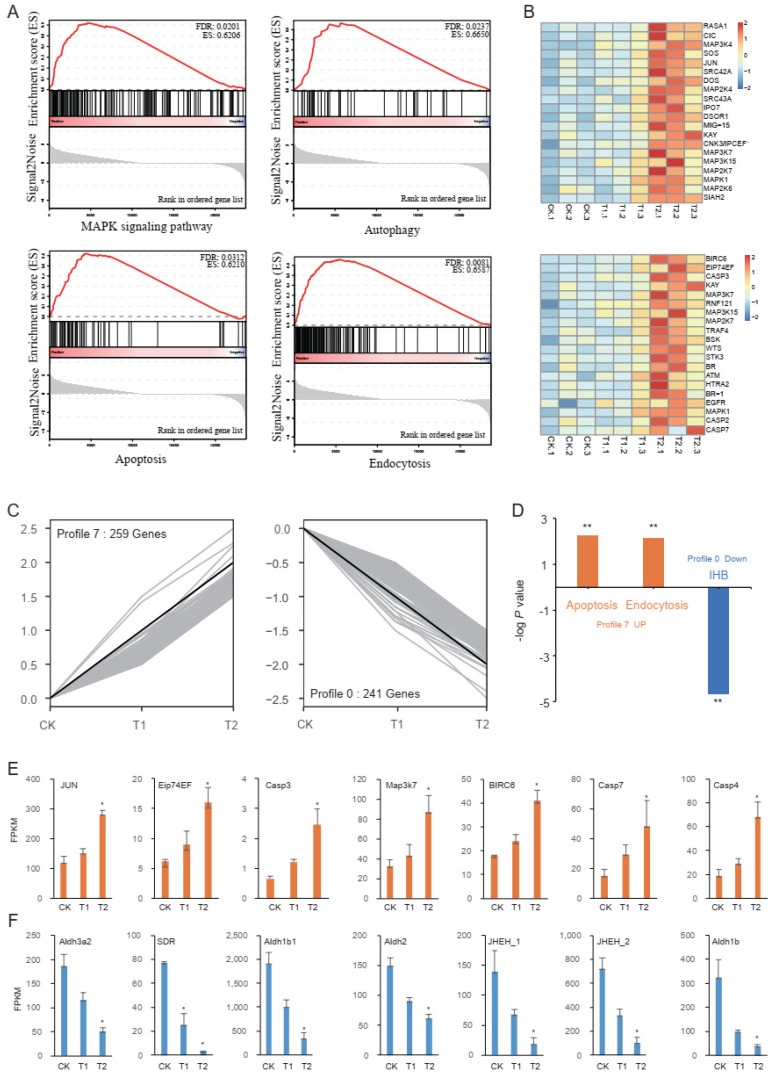
GSEA pathway analysis and trend analysis: (**A**) MAPK signaling, autophagy, apoptosis, and endocytosis pathways were significantly enriched in GSEA analysis; these pathways were upregulated, and FDR *q*-values are shown. (**B**) Heatmaps of important genes involved in the pathway of MAPK signaling (upper panel) and apoptosis (lower panel). (**C**) Significantly enriched trend analysis of profile 7 and profile 0. (**D**) The KEGG pathways enriched in profile 7 (upregulated) and profile 0 (downregulated); *y*-axis represents the −log *p*-value; ** *p* < 0.01. (**E**,**F**) The expression levels of candidate genes involved in the pathways of apoptosis (**E**) and insect hormone biosynthesis (**F**), * *p* < 0.05.

To further determine the differentially expressed pathways in feeding on the non-toxin-added artificial diet vs. the Vip3Aa diet in the RNA-Seq data, we performed GSEA between CK (control diet without toxin) and T2 (Table S5). Using a false discovery rate (FDR) *q*-value cutoff of 0.05, we found that a series of pathways were activated in Vip3Aa treatment, including the MAPK signaling pathway (FDR *q*-value < 0.05), autophagy pathway (FDR *q*-value < 0.05), apoptosis pathway (FDR *q*-value < 0.05), and endocytosis pathway (FDR *q*-value < 0.01) (Figure 2A). We speculated that apoptosis may be related to the MAPK signal pathway, since many common genes are involved in both pathways. Based on the enrichment scores, we identified the core enrichment genes in the MAPK signaling and apoptosis pathways (Figure 2B). We found that the expression levels of seven MAPK genes (MAPK3K4, MAP2K4, MAP3K7, MAP3K15, MAP2K7, MAPK1, and MAP2K6) were significantly induced in MAPK signaling pathway. In the apoptosis pathway, four MAPK genes (MAPK3K7, MAP3K15, MAP2K7, and MAPK1) were also significantly induced. Furthermore, three caspase genes (casp2, casp3, and casp7) were also significantly induced by Vip3Aa.

### 2.4. Trend Analysis

The DEGs from CK, T1, and T2 were clustered into eight profiles (Figure S1). In profile 7, all 259 genes were significantly upregulated with the treatments (Figure 2C). On the other hand, all 241 genes in profile 0 were significantly downregulated with the treatments (Figure 2C). To determine the biological functions of DEGs in profiles 0 and 7, KEGG analysis was carried out. A total of 13 pathways were enriched in profile 7, including the apoptosis (*p*-value < 0.01) and endocytosis (*p*-value < 0.01) pathways (Figure 2D). In profile 0, 30 pathways were significantly enriched, of which most were related to metabolism. The insect hormone biosynthesis pathway (*p*-value < 0.01), which plays important roles in various physiological activities of insects, was also significantly enriched in profile 0 (Figure 2D). In addition, we also found significant differences in the expression levels of some key candidate genes involved in the apoptosis and insect hormone biosynthesis pathways (Figure 2E,F).

### 2.5. Proteomic Profiling of S. frugiperda’s Response to Vip3Aa

**Figure 3:**
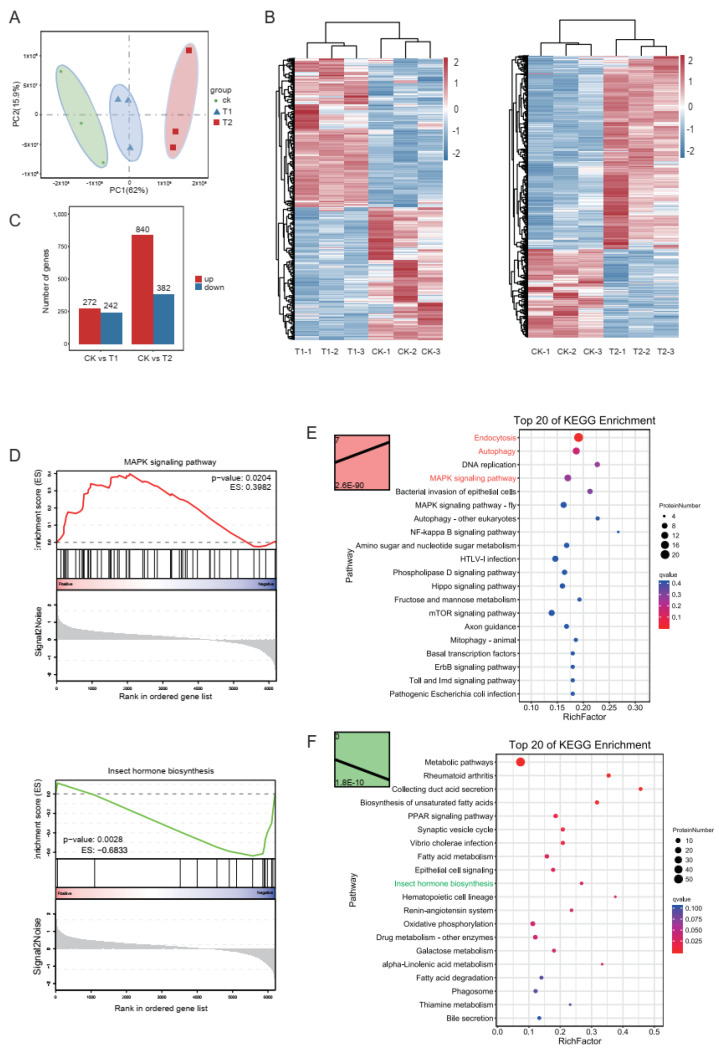
Proteomic analysis of Vip3a treatment: (**A**) PCA results of control (CK), Vip3Aa treatment 1 (T1), and Vip3Aa treatment 2 (T2). (**B**) Heatmaps of the expression of proteins. (**C**) The numbers of differentially expressed proteins. (**D**) MAPK signaling pathway and insect hormone biosynthesis pathway enriched in GSEA analysis. (**E**) Gene set of profile 7 was significantly enriched in trend analysis—the top 20 enriched KEGG pathways of differentially expressed proteins (DEPs) in profile 7; the bubble size represents the number of DEPs, while the color represents the *q*-value. (**F**) Gene set of profile 0 was significantly enriched in trend analysis—the top 20 enriched KEGG pathways of DEPs in profile 0; the bubble size represents the number of DEPs, while the color represent the *q*-value.

To ascertain the global changes in proteins regulated by Vip3Aa, the proteomes of the same midguts described earlier (three biological replicates) were analyzed using iTRAQ technology. Principal component analysis (PCA) showed that there were clear distinctions between these three treatments (Figure 3A). In total, 6207 proteins were identified, among which 514 (272 upregulated, 242 downregulated) and 1222 (840 upregulated and 382 downregulated) proteins showed significantly altered levels (fold change > 1.2, *p*-value < 0.05) between CK vs. T1 treatment and CK vs. T2 treatment, respectively (Figure 3B,C, Tables S6 and S7). To further determine the biological functions of the differentially expressed proteins (DEPs), we performed KEGG pathway analysis, GSEA analysis (Figure 3D), and trend analysis (Figure 3E,F). We found that MAPK signaling pathways were significantly enriched in both KEGG analysis of DEPs and trend analysis of profile 7 (upregulated in T1 and T2). In trend analysis of profile 7, the pathways of endocytosis and autophagy were also enriched. Meanwhile, the pathway of insect hormone biosynthesis was significantly enriched in profile 0 (downregulated in T1 and T2), and was also enriched in KEGG analysis of DEPs and GSEA analysis (Figure 3F). The functional analysis of proteomics was consistent with that of transcriptomics, mainly enriched in signal transduction, hormone regulation, apoptosis, and autophagy.

### 2.6. Correlation Analysis the Transcriptomic and Proteomic Data

**Figure 4:**
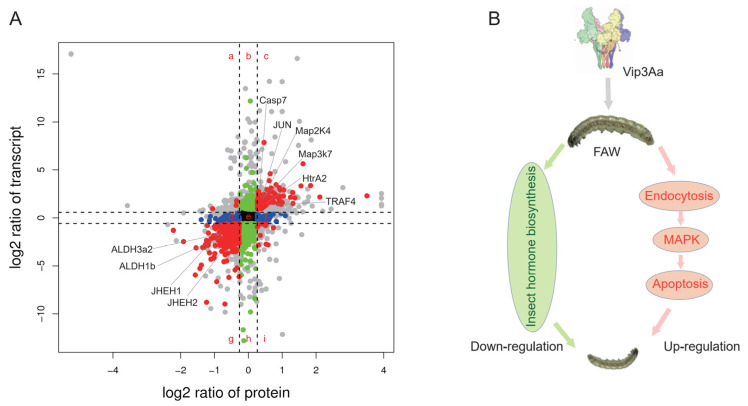
Correlation analysis between genes and proteins: (**A**) The expression levels of genes and proteins in the control and Vip3Aa treatment groups were plotted in the log_2_ scale, with the *y-* and *x*-axes indicating the relative gene and protein levels, respectively; red: the expression levels of genes and proteins were significantly changed; blue: significant changes in protein expression; green: significant changes in gene expression. (**B**) Summary of the responses of *S. frugiperda* to Vip3Aa; Vip3Aa could induce the upregulation of the endocytosis and MAPK pathways, and then activated the caspase-mediated apoptosis pathway, leading to insect death; meanwhile, Vip3Aa induced the downregulation of the insect hormone biosynthesis pathway, suppressing the growth of the insects.

Combined analysis of RNA-Seq and proteomic data provides an opportunity to investigate the relationships between transcriptional profiles and the translational profile. Here, we examined the correlation between mRNA and protein expression in CK vs. T2 treatment. A Venn diagram was used to display the number of genes in transcriptomics and the number of proteins in proteomics. A total of 6084 genes were commonly detected. The distribution of the mRNA:protein ratios of those commonly detected genes was shown by a scatterplot analysis of the log_2_-tranformed ratios. We grouped the mRNA:protein ratios into nine groups based on the following patterns: groups a and i—the directions of mRNA and protein changes were opposite; groups b and h—protein levels were essentially unchanged, whereas the mRNA levels were induced or repressed; groups d and f—mRNA levels were essentially unchanged, whereas the protein levels were induced or repressed; group e—mRNA and protein were unchanged; groups c and g—the mRNA and protein levels showed the same changes (Figure 4A). Approximately 76.9% of genes and proteins showed the same directions, indicating that most proteins showed a positive correlation with their mRNAs in expression patterns. On the basis of this gene set, we analyzed the function of group c, where mRNA and protein expression level were upregulated, and group g, where mRNA and protein expression level were downregulated. In group c, the most significantly enriched pathway was the apoptosis pathway (*p*-value < 0.01), followed by the endocytosis and MAPK signaling pathways. Key genes involved in those pathways—such as MAP2K4, MAP3K7, casp7, JUN, HtrA2, and TRAF4—are shown in Figure 4A (Table S8). In group g, a total of 30 pathways were enriched, most of which pertained to metabolic pathways (Table S8). Notably, the insect hormone biosynthesis pathway was significantly enriched in group g, and key genes involved in this pathway are also shown in Figure 4A.

## 3. Discussion

A better understanding of the mechanisms of insects’ responses after exposure to Bt toxins will broaden our knowledge of the impact on larvae mediated by Bt toxins, helping us to better understand the mode of action of Bt toxins. In lepidopteran insects, the midgut tissue plays important roles in feeding, and is the principal tissue affected by Bt toxins [3]. In the present study, the gene expression profiles and protein expression profiles of midguts treated with Vip3Aa were analyzed using transcriptomics and proteomics, respectively. Firstly, we obtained a comprehensive pattern of gene and protein changes. Based on these differentially expressed genes and proteins, we performed pathway analysis, GSEA analysis, trend analysis, and transcriptomic and proteomic correlation analysis. The results indicated that Vip3Aa could induce the apoptosis pathway, with the involvement of the MAPK signaling and endocytosis pathways. On the other hand, Vip3Aa could significantly downregulate the insect hormone biosynthesis pathway. Moreover, we also identified some important candidate genes involved in those important pathways.

In this study, we identified 873 significant differentially expressed genes under the treatment with 10 μg/g Vip3Aa, whereas 2227 significant differentially expressed genes were identified under the 50 μg/g Vip3Aa treatment, suggesting that the host response increased with the increase in Vip3Aa concentration. Similar to previous reports, the downregulated genes were mainly enriched in metabolic pathways [19]. We found that the insect hormone biosynthesis pathway, which plays important roles in insect development, was significantly downregulated. Insect hormones were reported that can orchestrate the expression of Bt receptors via the MAPK signaling pathway, and are involved in Bt resistance in *Plutella xylostella* [20]. When the concentration of Vip3Aa increased, some new pathways were enriched, including the apoptosis pathway. The reported receptors of Vip3Aa—SRC and FGFR—were both associated with apoptosis [10,11]. For SRC, thrombospondin-1 activated SR-B2, and then SR-B2 triggered downstream signaling via the MAPK pathway and caspase pathway, with increased apoptosis in vascular cells [21]. For FGFR, it has been reported that FGFR is associated with apoptosis in lung cancer cell lines and murine models [22,23].

Importantly, GSEA of differentially expressed genes identified several significant pathways (*p* < 0.05) that were activated in the treatment with 50 μg/g Vip3Aa—most notably, the MAPK signaling, autophagy, apoptosis, and endocytosis pathways. Considerable prior work has shown all of these pathways to be associated with Bt toxins [10,20,24]. Next, we analyzed the differentially expressed genes involved in the MAPK signaling and apoptosis pathways, and found that multiple genes were commonly involved in both pathways. In the apoptosis pathway, we identified 4 MAPK genes and 3 caspase genes. Combined with previous reports, we speculated that Vip3Aa may activate caspase-mediated apoptosis via the MAPK signaling pathway. Moreover, in order to obtain more information from RNA-Seq, we also performed trend analysis. Functional analysis of two significantly enriched profiles (*p*-value < 0.05) indicated that the apoptosis and endocytosis pathways were enriched in profile 7, while the insect hormone synthesis pathway was enriched in profile 0. The genes involved in these pathways were clearly shown to be dose-dependent.

The expression levels of mRNAs may not fully represent the expression levels of proteins, due to the post-translational modifications. Proteomic analysis was also performed in order to analyze the response of midguts to Vip3Aa in this study. In total, we identified 6207 proteins, of which 5151 proteins had annotation information. This protein set may provide information for higher quality genome annotation. Similar to the results of RNA-Seq, the number of differentially expressed proteins was also increased with the increase in Vip3Aa concentration. Next, we performed functional analysis based on the differentially expressed proteins. Pathway analysis, GSEA, and trend analysis results showed that the MAPK signaling, endocytosis, and autophagy pathways were induced, whereas the insect hormone biosynthesis pathway was downregulated. These results are consistent with the results of RNA-Seq. In addition, integrative analysis of RNA-Seq and proteomic data clustered the genes into nine groups, according to the expression patterns of genes and proteins. It is interesting to note that the genes involved in MAPK signaling, endocytosis, and apoptosis were enriched in group c, in which proteins and transcripts increased in parallel. The insect hormone synthesis pathway was enriched in group g, in which proteins and transcripts decreased in parallel. Combining the results of transcriptomics and proteomics, we proposed a model for larval response to Vip3Aa, involving induction of the endocytosis and MAPK pathways, leading to apoptosis via caspase, while simultaneously decreasing the insect hormone synthesis pathway (Figure 4B).

In conclusion, our results show that exposure of *S. frugiperda* larvae to sublethal concentrations of Vip3Aa activates different insect response pathways, including induction of the endocytosis and MAPK signaling pathways, and triggers the apoptosis pathway, leading to apoptotic cell death. The results of the present study will promote our understanding of the host response process to Vip3Aa, and help us to shed light on the mode of action of Vip3A toxins.

## 4. Materials and Methods

### 4.1. Insects

*S. frugiperda* strain DH19 was collected from a corn field in Dehong (Ruli, Yunnan Province, China) in 2019 and reared on an artificial diet (main ingredients: corn and soybean), without exposure to pesticides [25]. Larvae were reared at 27 ± 2 °C, relative humidity (RH) of 75 ± 10%, and a photoperiod of 14L:10D. For adults, 10% sugar solution was supplied to replenish energy.

### 4.2. Treatment by Vip3Aa

The Vip3Aa protoxins used in the study were provided by the institute of Plant Protection, Chinese Academy of Agricultural Sciences. According to the previous bioassay results, two different concentrations of Vip3Aa were selected for bioassay. Third-instar larvae (24 × 3) were fed with 10 μg/g or 50 μg/g of Vip3Aa toxin, and their average relative growth rates were compared with those of larvae fed with a control diet. After 8 h of starvation, the larvae were weighed and recorded as M1, and then transferred to the diet. Larvae were re-weighed 72 h later and recorded as M2. Relative growth rate was calculated as [(M2-M1)/M1]. The midguts were excised from the treatment instars and stored at −80 °C for RNA-Seq and proteome sequencing.

### 4.3. RNA Extraction and cDNA Library Construction

Total RNA of midguts was extracted using TRIzol reagent kits (Invitrogen, Waltham, MA, USA), following the manufacturer’s protocol. After extraction, the quality of RNA was assessed on an Agilent 2100 Bioanalyzer, and checked using RNase-free agarose gel electrophoresis. Then, sequencing libraries were generated using the NEBNex1 Ultra RNA Library Prep Kit (NEB, Ipswich, MA, USA). A total of 9 sequencing libraries were constructed (three treatments, with three biological replicates). The libraries were sequenced on the HiSeq 2500 sequencing platform at Gene Denovo Biotechnology Co. (Guangzhou, China).

### 4.4. The Analysis of RNA-seq Data

After removing reads containing adapters, low-quality reads, and reads containing poly-N, the clean reads were obtained. rRNAs were removed with the Bowtie program (version 2.2.8) [26]. The clean data were then mapped to the reference genome (https://www.ncbi.nlm.nih.gov/Traces/wgs/?val=WUTJ02, accessed on 21 May 2021) using HISAT (version 2.2.4) [27]. The expression levels of genes were estimated by RSEM and normalized using the fragments per kilobase of transcripts per million mapped reads (FPKM). Differentially expressed genes (DEGs) (FDR < 0.05 and |log2 (fold-change)| ≥ 1.5) were identified using the edgeR package (version 3.12.1) [28]. Gene Ontology (GO) analysis was performed with the goseq R package, while KEGG pathway enrichment was conducted using the OmicShare tools (www.omicshare.com/tools, accessed on 5 July 2021).

### 4.5. Gene Set Enrichment Analysis (GSEA) and Trend Analysis

Gene set enrichment analysis was performed using GSEA software [29]. The GSEA was used to identify whether a set of genes in specific pathways showed significant differences in different groups. Briefly, we input gene expression matrices and ranked genes by the signal-to-noise normalization method. Then, enrichment scores and *p*-values were calculated for default parameters. To analyze the expression patterns of DEGs, the expression data of each sample were normalized, and then clustered using STEM software [30]. The clustered profiles with *p* < 0.05 were considered to be significant profiles. The DEGs in each profile were subjected to KEGG pathway enrichment analysis.

### 4.6. iTRAQ-Based Proteome Analysis

Total protein of midguts was extracted using the Tissue Protein Extraction Kit (Cwbiotech Co., Ltd., Beijing, China), according to the manufacturer’s protocol. After trypsin digestion (modified trypsin, Promega, Madison, at a substrate/enzyme ratio of 50:1 (*w*/*w*) at 37 °C for 16 h), the resultant peptides were dried using vacuum centrifugation. iTRAQ labeling was performed using an iTRAQ Reagent 8PLEX Multiplex Kit (Applied Biosystems, US), according to the manufacturer’s protocol. The detail results of nano-HPLC–MS/MS analysis were similar to previous reports [31]. Raw data were processed and analyzed via Spectronaut X, with default parameters. We set 1% as the *q*-value (FDR) cutoff for precursors and protein levels. Decoy generation was set to mutated, which is similar to scrambled, but only applies a random number of AA position swaps (min = 2, max = length/2). All selected precursors passing the filters were used for quantification. After Student’s *t*-Test, 1.2-fold change and *p*-value < 0.05 was set as the threshold to screen differentially expressed proteins. Proteins were annotated against the KEGG database, and significant pathways were examined for DEPs with *p*-values < 0.05.

## Data Availability

The RNA-seq sequencing reads are available for download at Sequence Read Archive (SRA) NCBI accession number PRJNA783715.

## Supplementary Materials

The following supporting information can be downloaded at https://www.mdpi.com/article/10.3390/toxins14010055/s1: Table S1: The list of differentially expressed genes in comparison of WT vs. T1; Table S2: The list of differentially expressed genes in comparison of WT vs. T2; Table S3: The detail of KEGG pathways enriched in comparison of group CK vs. T1; Table S4: The detail of KEGG pathways enriched in comparison of group CK vs. T2; Table S5: Genes involved in pathways analyzed by GSEA; Table S6: The list of differentially expressed proteins in comparison of WT vs. T1; Table S7: The list of differentially expressed proteins in comparison of WT vs. T2; Table S8: The detailed information of enriched KEGG pathways in groups c and g; Figure S1: The clustered profiles analyzed by trend analysis.
