# Supplementary material for: An Integrative Analysis of Transcriptomics and Proteomics Reveals Novel Insights into the Response in the Midgut of Spodoptera frugiperda Larvae to Vip3Aa
Source: Toxins (Basel). 2022 Jan 13;14(1):55. doi: 10.3390/toxins14010055 (PMC8781260; doi:10.3390/toxins14010055)
Supplement: Supplementary file 1 [file toxins-14-00055-s001.zip › toxins-1509842-SI-Figure S1.pdf]

An Integrative Analysis of Transcriptomic and Proteomic Reveals Novel Insights Into the Response in the Midgut of *Spodoptera frugiperda* Larvae to Vip3Aa

Minghui Jin, Yinxue Shan, Yan Peng, Ping Wang, Qi Li, Songmiao Yu, Lei Zhang and Yutao Xiao

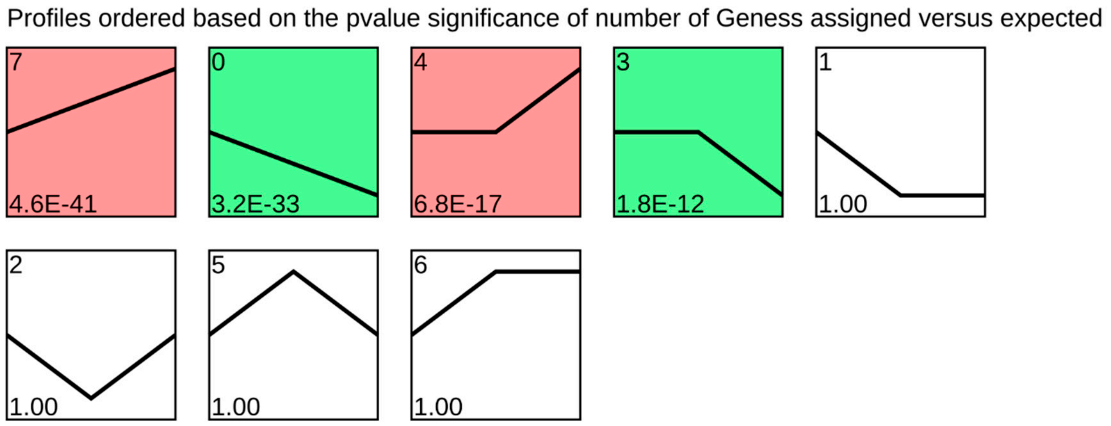

Figure S1. The clustered profiles analyzed by trend analysis.
